# Trait divergence and habitat specialization in tropical floodplain forests trees

**DOI:** 10.1371/journal.pone.0212232

**Published:** 2019-02-15

**Authors:** Gisele Biem Mori, Juliana Schietti, Lourens Poorter, Maria Teresa Fernandez Piedade

**Affiliations:** 1 Instituto Nacional de Pesquisas da Amazônia, INPA, Manaus, Amazonas, Brazil; 2 Forest Ecology and Forest Management Group, Wageningen University & Research, WUR, Wageningen, The Netherlands; UNAM, MEXICO

## Abstract

Habitat heterogeneity of tropical forests is thought to lead to specialization in plants and contribute to the high diversity of tree species in Amazonia. One prediction of habitat specialization is that species specialized for resource-rich habitats will have traits associated with high resource acquisition and fast growth while species specialized for resource-poor habitats will have traits associated with high resource conservation and persistence but slow growth. We tested this idea for seven genera and for twelve families from nutrient-rich white-water floodplain forest (várzea) and nutrient-poor black-water (igapó) floodplain forest. We measured 11 traits that are important for the carbon and nutrient balance of the trees, and compared trait variation between habitat types (white- and black-water forests), and the effect of habitat and genus/family on trait divergence. Functional traits of congeneric species differed between habitat types, where white-water forest species invested in resource acquisition and productive tissues, whereas black-water forest species invested in resource conservation and persistent tissues. Habitat specialization is leading to the differentiation of floodplain tree species of white-water and black-water forests, thus contributing to a high diversity of plant species in floodplain forests.

## Introduction

Habitat filtering is a key mechanism underlying assembly of plant communities as it determinates whether species are able to establish, grow and reproduce under specific abiotic conditions [1]. Species are filtered out based on their functional traits, and habitat filtering usually explains the co-occurrence of species under similar abiotic conditions as a result of shared traits and ecological similarity. Ecological similarity though, is often caused by phylogenetic relatedness between species [2,3], meaning that related species tend to have more similar trait values. Therefore, comparing phenotypic differences in plant traits between related species is a simple way to control for phylogenetic relatedness, and to test whether different habitat selecting pressures have led to species trait differentiation [4,5].

Habitat heterogeneity is thought to be an important cause of tree species diversity in Amazonian forests [6–8]. Few Amazonian tree species are dominant while many are rare [9], and it is assumed that the majority of coexisting species are habitat generalists [10]. Yet, the high turnover of tree species across local environmental gradients suggests that species do specialize to specific habitat conditions, thus promoting species diversity and coexistence [7,11,12].

Many plant traits diverge as an adaptation to environmental conditions, for instance, topography, soil nutrients and water availability, leading to species preference for different habitat types [13–16]. The occurrence of phylogenetically closely related species in contrasting habitats, and the co-occurrence of unrelated species in the same habitat with similar abiotic conditions, indicates that species have specialized for different habitats [8]. Trait divergence between closely related species that occur in different habitats is therefore an indication of how habitat specialization occurs [4,17].

Rather than single traits, it is a suite of different traits that allow species to specialize for a certain habitat condition [4,14]. In combination, this suite of traits forms a plants strategy. For example, large leaves, high specific leaf area and high leaf nutrient concentrations are usually associated with fast-growth which is advantageous in high-resource environments, whereas low specific leaf area and high leaf dry matter content reflect investment in strong and persistent tissues that increase nutrient conservation, water use efficiency and plant survival in low-resource environments [18–20]. Habitat specialization is, therefore, an important ecological process that leads to the development of different ecological strategies in plant communities, thus contributing to species diversity [21–23].

Amazonian floodplain forests provide a unique ecosystem to evaluate the influence of habitat specialization. These forests face seasonal flooding dynamics [24,25] which allows only species with specific traits and strategies to survive under these anoxic conditions [26–28]. One important strategy to deal with flooding is to slow down the metabolism to save energy and resources, which is reflected in the sclerophyllous leaves, lower plant height and diameter and higher wood density. Another strategy, is the development of lenticels in roots and trunks, which are responsible for the gas exchange in flooded soils that have low oxygen available [26,28]. Different floodplain forest types are recognized based on their contrasting soil nutrient availability, the nutrient-rich white-water forests (várzea) and the nutrient-poor black-water forests (igapó; [24,29]). Although many species occur in floodplain forests, white- and black-water forests share only 30% of the species [30]. Because of the low floristic similarity between white- and black-water forests, habitat specialization is likely to be the main process underlying the floristic differences between these two forest types.

In this study, we evaluated how functional traits diverge between congeneric tree species of two floodplain forests of Central Amazonia that have contrasting soil resource conditions [31]. We addressed the following questions and hypotheses: (1) Do functional traits diverge between congeneric species living in different floodplain forest with different soil types? We hypothesized that congeneric species that occur in nutrient-rich white-water forests will have acquisitive trait values (i.e., higher specific leaf area and leaf nutrients) and fast-grow, whereas congeners inhabiting nutrient-poor black-water forests will have conservative trait values (i.e., higher wood density and leaf dry matter content) that increase persistence under nutrient poor conditions but lead to slow-growth. Variation in trait values will be lower in black-water species compared to white-water species because strong limitation of nutrient resources [31]; (2) Can variation in trait values between congeneric species be attributed to differences among genera or to forest types? We hypothesized that habitat (forest types) will be more important for trait differentiation, and will lead to the divergence of traits between white- and black-water forest species within the same genus.

## Material and methods

### Study sites and sample design

Research was carried out in Central Amazonia which is characterized by a hot and humid tropical climate. Mean annual temperature is 26.6 °C. Mean annual precipitation is 2,100 mm/y, with a wet season occurring from December to April and a dry season from June to October. White-water forests have higher soil water retention capacity (higher silt and clay concentrations), and higher macro- and micro-nutrient concentration, especially calcium, manganese and base cations. Black-water forests have sandy soils and low macro- and micro-nutrient concentrations compared to white-water forests [31,32].

Trees from white-water and black-water forests species were sampled in 25x25m plots previously established and inventoried in two reserves, the Mamirauá Sustainable Development Reserve (RDS Mamirauá) and the Uatumã Sustainable Development Reserve (RDS Uatumã). The flooding regime varies from 2 to 5 meters, during 180–240 days in the white-water forests; and from 1 to 3 meters, during 50–100 days in black-water forests. The RDS Mamirauá (2°51’S 64°55’W) has an area of 124,000 km^2^ of white-water floodplain forests, and is adjacent to the Japurá and Solimões river and the Auati-paranã channel [33] (Wittmann et al., 2002). The Uatumã RDS (1°48′S, 59°15′W) has an area of 4,244.300 km^2^, and is located between Itapiranga and São Sebastião do Uatumã cities [34]. Field work was carried out under collecting permits 015/2016-DEMUC/SEMA and SISBIO 52109–1, provided by Departamento de Mudanças Climáticas e Gestão de Unidades de Conservação da Secretaria Estadual de Meio Ambiente of Amazonas State and Instituto Chico Mendes de Conservação da Biodiversidade, respectively.

To evaluate whether habitat specialization occurs, we carried out our analysis at the genus and also at the family level, to verify if an older evolutionary split is causing specialization. We selected seven genera that occur in both white- and black-water forests along the flooding gradient that have at least 5 individuals per genus in each forest type, to estimate the average trait value of a genus (Table 1). The threshold of 5 individuals was chosen because of the low co-occurrence of species between these forest types [30]. The individuals within a genus belong from 1 to 4 species. In total we sampled 70 individuals (35 individuals per forest type, and 5 individuals per genus). These individuals belonged to 29 species, with 14 species occurring only in white-water forest, 10 species occurring only in black-water forest, and 5 species occurring in both forest types (S1 Table). The criteria for family selection was the same as for genus (i.e. 5 individuals per family per forest type), leading to a total of 12 families that occurred in both forest types (Table 1). We sampled 120 individuals (50 individuals per forest type) and 54 genera, with 22 genera occurring only in white-water forest, 14 genera occurring only in black-water forest, with 18 occurring in both forest types (S1 Table).

**Table 1:** Seven genera and 12 families of the study. The columns indicate the genus/family, and the species/genus number belonging to each genus/family in white-water and black-water forests. The number of individuals sampled for each genus/family per forest type is 5 (n = 5).

Phylogenetic level	species number	
Genus	White-water forest	Black-water forest
*Eschweilera* (Lecythidaceae)	3	3
*Guatteria* (Annonaceae)	2	1
*Licania* (Chrysobalanaceae)	3	5
*Mouriri* (Melastomataceae)	2	2
*Ocotea* (Lauraceae)	3	2
*Pouteria* (Sapotaceae)	2	1
*Zygia* (Fabaceae)	4	1
Family	genus number	
Annonaceae	5	1
Apocynaceae	3	2
Chrysobalanaceae	3	2
Euphorbiaceae	5	4
Fabaceae	5	5
Lauraceae	1	1
Lecythidaceae	4	3
Malvaceae	5	1
Melastomataceae	1	2
Moraceae	4	2
Myrtaceae	2	5
Sapotaceae	3	4

### Functional traits

For each tree we measured eleven functional traits that are related to resource acquisition and growth (S2 and S3 Tables). The traits were Leaf area—LA (cm^2^), Specific leaf area—SLA (cm^2^.g^-1^), Leaf dry matter content—LDMC (g.g^-1^), Chlorophyll content—Chl (SPAD units), Leaf nitrogen—N (g.kg^-1^), Leaf phosphorus—P (g.kg^-1^), Leaf Potassium—K (g.kg^-1^), Leaf Calcium—Ca (g.kg^-1^), Branch wood density—BWD (g.cm^-3^), Lenticel density—LD (count in 10 cm^-2^), and the Height:diameter ratio—HDR (m.cm^-1^ of the tree. Measurements were made following standardized trait protocols [35] (Table 2). Leaf traits were measured in 5 sun-exposed leaves per individual. Chlorophyll content was measured with a chlorophyll meter (SPAD-502) on 3 sun-exposed leaves. Lenticel density was measured in the trunk of the trees using a 10 cm^2^ templet. To reduce sampling effort and tree damage, we measured wood density at the branch level instead of the trunk level [36]. BWD was measured in one branch per individual (bark included) using the water displacement method [36]. The measurements of LA, SLA, LDMC, Chl, BWD, LD and HDR were conducted in the field and leaf nutrient analysis was done by Empresa Brasileira de Pesquisa e Agropecuária (Embrapa, Manaus, Brazil).

**Table 2:** Overview of the plant functional traits included in this study, with the trait name, abbreviations, units and their function.

Plant functional trait	Function related	Reference
Leaf area, LA (cm^2^)	Light intercepting area, respiration, transpiration, gas exchange	[37]
Specific leaf area, SLA (cm^2^.g^-1^)	Light capture economics, net assimilation rate, relative growth rate, leaf life span, photosynthetic capacity	[38,39]
Chlorophyll content, Chl (SPAD units)	Light uptake efficiency, photosynthetic rates	[39]
Leaf dry matter content, LDMC (g.g^-1^)	Construction costs, nutrient retention, resistance against herbivory and physical damage, drought resistance	[37,38]
Leaf nitrogen (N) and phosphorus (P) concentration (g.kg^-1^)	Photosynthetic rates, CO_2_ assimilation, leaf nutrients levels	[19,20]
Leaf potassium, K (g.kg^-1^)	Stomatal control, turgor provision and water homeostasis	[40]
Leaf Calcium, Ca (g.kg^-1^)	Cellwall structure	[40]
Branch wood density, BWD (g.cm^-3^)	Construction costs, growth rate, pathogen resistance, mortality rate	[36,41]
Lenticel density, LD (count of lenticels/10 cm^-2^)	Oxygen uptake, gas exchange	[26]
Height:diameter ratio of the plant, HDR (m.cm^-1^)	Stability, competitive strength	[42,43]

### Data analysis

To analyse how functional traits values vary within habitat types we calculated the mean value for each genera, and also the coefficient of variation for each forest type as the standard deviation multiplied by 100. To evaluate how functional traits vary between congeners in different habitat types (white- and black-water forests) we made for each trait a graph where the trait mean values of white- and black-water genera were paired. To evaluate if functional trait values diverge between forest types and genera we used a Linear mixed model for each functional trait, using forest type as a fixed effect and genus as a random effect [44]. For each trait, we compared models considering both habitat and genus (model_1_) and considering only habitat (model_2_) using likelihood ratio test, which indicates if models differ with the addition of genus effect [45]. To assess the importance of genus for trait variation we calculated the marginal and conditional R squared of each linear mixed model. The marginal R^2^ describes the proportion of variance explained by the fixed effect (habitat), while the conditional R^2^ describes the proportion of variance explained by both fixed and random effect (habitat + genus; [46]). When the conditional R^2^ value is similar to marginal R^2^, it indicates that the factor genus is not affecting plant traits. To verify whether an older evolutionary split would show the same pattern, we carried out the same analysis at the family level and compared the results. All analyses were carried out using the R platform [47].

## Results

### Traits divergence between forest types

**Fig 1:**
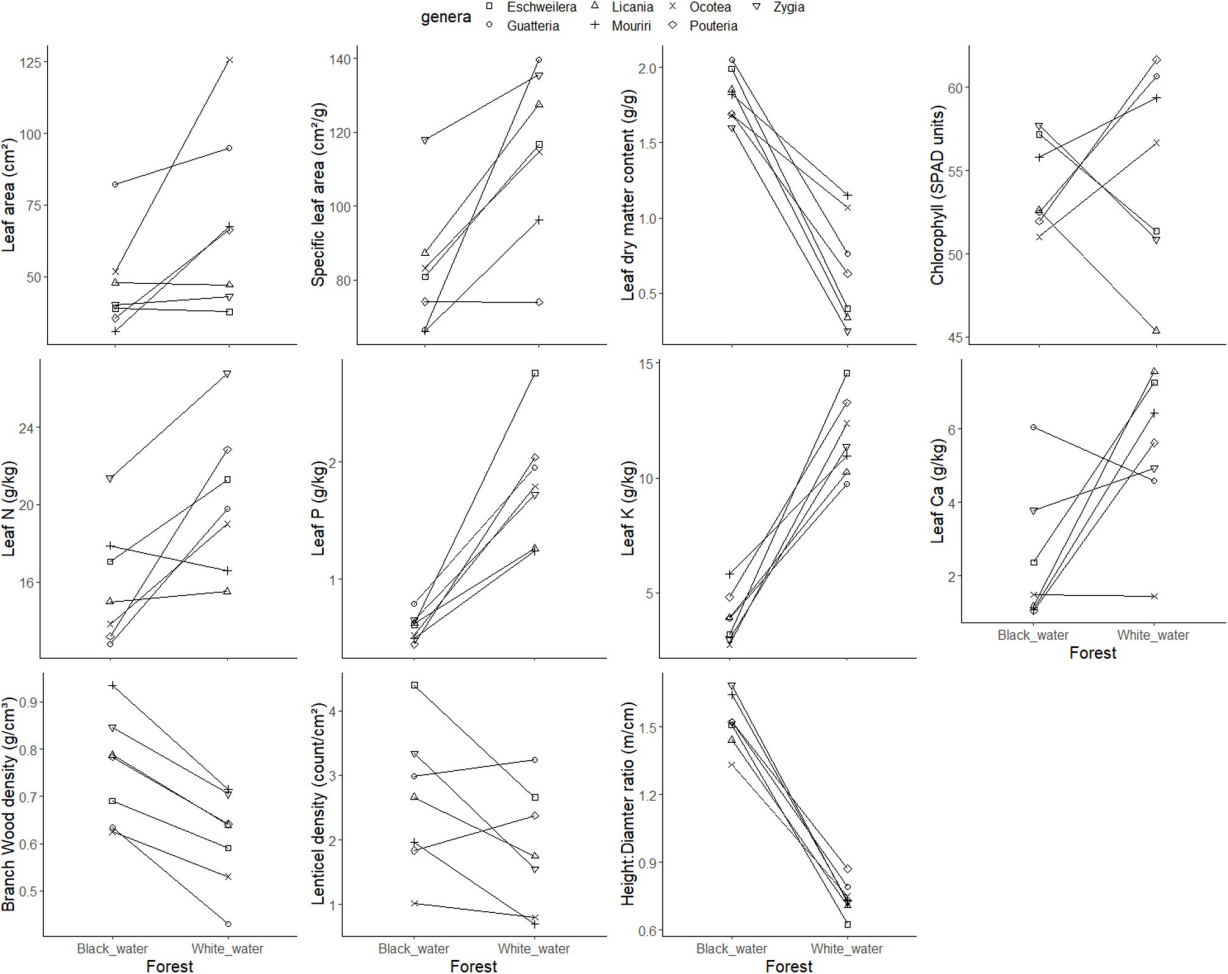
Mean values of leaf area, specific leaf area, leaf dry matter content, leaf chlorophyll, leaf nitrogen (N), leaf phosphorus (P), leaf potassium (K), leaf calcium (Ca), branch-wood density, lenticel density and height:diameter ratio for seven pairs of congeneric species of white-water and black-water forests. Lines connect the congeneric pairs. Mean values were based on 5 individuals per genus and forest type.

**Fig 2:**
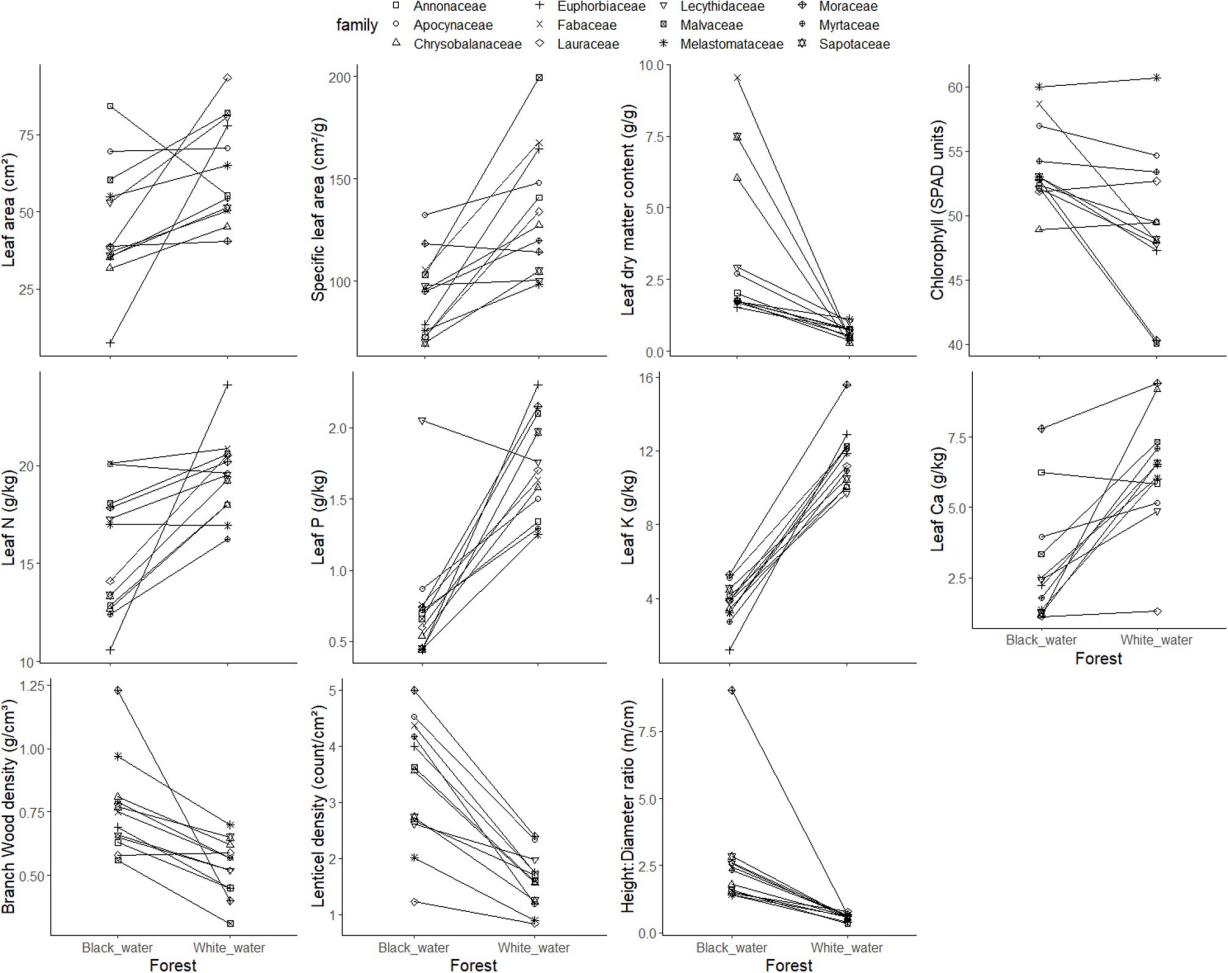
Mean values of leaf area, specific leaf area, leaf dry matter content, leaf chlorophyll, leaf nitrogen (N), leaf phosphorus (P), leaf potassium (K), leaf calcium (Ca), branch-wood density, lenticel density and height:diameter ratio for twelve pairs of families of white-water and black-water forests. Lines connect the family pairs. Mean values are based on 5 individuals per family and forest type.

All traits diverged between white- and black-water congeneric tree species (genus level comparison), except leaf chlorophyll content. White-water species had, on average, 1.2–3.0 times higher values of leaf area, specific leaf area and leaf nutrient concentrations, than congeneric black-water species. In contrast, black-water congeneric species had 1.2–2.7 times higher values of leaf dry matter content, branch wood density, lenticel density and height:diameter ratio (Table 3 and Fig 1). At the family level, the same traits had higher values for white-water or for black-water, maintaining the same patterns of trait variation for comparisons at an older phylogenetic level (family) as for comparisons at a more recent phylogenetic level (genus) (Table 4 and Fig 2). Frequently, trait variation among species within forest type was higher in the white-water forest than in the black-water forest, both when species comparisons were made at the genus level and the family level, indicated by the coefficient of variation (Tables 3 and 4). For some traits (especially leaf potassium and calcium), trait variation was larger in black-water forest than in white-water forest.

**Table 3:** Mean values of 11 functional traits of seven genera in white-water and black-water forests. The columns indicate trait mean values per genus: leaf area (LA), specific leaf area (SLA), leaf dry matter content (LDMC), leaf nitrogen (N), leaf phosphorus (P), leaf potassium (K), leaf calcium (Ca), branch-wood density (BWD), lenticel density (LD), height:diameter ratio (HDR). Trait mean values are based on 5 individuals per genera and coefficient of variation (*CV*) is based on all 33 species per forest type (see Table 2 for trait units).

Forest type	Genus level	LA	SLA	LDMC	Chl	N	P	K	Ca	BWD	LD	HDR
White-water	*Guatteria*	94.8	139.7	0.8	60.6	19.8	2.0	9.7	4.6	0.4	3.2	0.8
	*Licania*	47.0	127.5	0.3	45.4	15.5	1.3	10.3	7.6	0.6	1.7	0.7
	*Zygia*	43.1	135.5	0.3	50.9	26.8	1.7	11.4	4.9	0.7	1.6	0.7
	*Ocotea*	125.7	114.7	1.1	56.7	19.0	1.8	12.4	1.5	0.5	0.8	0.7
	*Eschweilera*	37.7	116.7	0.4	51.4	21.3	2.8	14.6	7.3	0.6	2.7	0.6
	*Mouriri*	67.4	96.2	1.2	59.4	16.6	1.2	11.0	6.4	0.7	0.7	0.7
	*Pouteria*	66.3	74.0	0.6	61.7	22.8	2.0	13.3	5.6	0.6	2.4	0.9
	mean	68.9	114.9	0.7	55.1	20.3	1.8	11.8	5.4	0.6	1.9	0.7
	*CV*	65	26	78	14	37	62	36	65	18	100	25
Black-water	*Guatteria*	82.0	66.4	2.1	52.5	12.8	0.8	3.9	6.0	0.6	3.0	1.5
	*Licania*	47.8	87.2	1.9	52.6	15.0	0.6	3.9	1.2	0.8	2.7	1.4
	*Zygia*	40.2	118.0	1.6	57.7	21.4	0.7	3.0	3.8	0.8	3.3	1.7
	*Ocotea*	51.7	83.2	1.7	51.0	13.8	0.5	2.8	1.5	0.6	1.0	1.3
	*Eschweilera*	38.9	80.8	2.0	57.2	17.1	0.6	3.2	2.4	0.7	4.4	1.5
	*Mouriri*	31.0	66.2	1.8	55.8	17.9	0.5	5.8	1.1	0.9	2.0	1.6
	*Pouteria*	35.5	74.1	1.7	52.0	13.2	0.4	4.8	1.1	0.8	1.8	1.5
	mean	46.7	82.3	1.8	54.1	15.9	0.6	3.9	2.4	0.8	2.6	1.5
	*CV*	45	31	13	10	23	36	40	89	15	78	25

**Table 4:** Mean values of 11 functional traits of twelve families in white-water and black-water forests. The columns indicate trait mean values per family: leaf area (LA), specific leaf area (SLA), leaf dry matter content (LDMC), leaf nitrogen (N), leaf phosphorus (P), leaf potassium (K), leaf calcium (Ca), branch-wood density (BWD), lenticel density (LD), height:diameter ratio (HDR). Trait mean values are based on 5 individuals per family and coefficient of variation (*CV*) is based on all 54 genera per forest type (see Table 2 for trait units).

Forest type	Family	LA	SLA	LDMC	Chl	N	P	K	Ca	BWD	LD	HDR
White-water	Annonaceae	55.3	140.8	0.5	49.5	18.0	1.3	10.0	5.8	0.5	1.7	0.6
	Apocynaceae	70.7	148.0	0.8	54.7	19.6	1.5	12.2	5.2	0.5	2.3	0.6
	Chrysobalanaceae	45.3	127.3	0.3	49.5	18.0	1.6	10.1	9.2	0.6	1.6	0.6
	Euphorbiaceae	78.0	164.7	0.8	47.3	24.1	2.3	12.9	6.5	0.5	1.8	0.4
	Fabaceae	50.4	167.7	0.5	48.0	20.9	1.6	12.2	6.1	0.6	1.8	0.7
	Lauraceae	93.5	133.9	0.8	52.7	20.5	1.7	11.2	1.3	0.6	0.8	0.8
	Lecythidaceae	80.7	100.4	1.1	47.8	19.5	1.8	9.7	4.9	0.5	2.0	0.5
	Malvaceae	82.0	199.4	0.8	40.1	20.6	2.1	12.3	7.3	0.3	1.6	0.3
	Melastomataceae	65.0	98.5	1.1	60.7	16.9	1.3	11.9	6.0	0.7	0.9	0.6
	Moraceae	40.5	114.4	0.4	40.3	20.2	2.2	15.6	9.4	0.4	2.4	0.7
	Myrtaceae	54.2	119.8	0.6	53.4	16.3	1.3	10.9	7.1	0.6	1.2	0.6
	Sapotaceae	51.3	104.9	0.5	48.2	19.3	2.0	10.5	6.6	0.7	1.3	0.6
	mean-value	65.3	134.7	0.6	52.2	20.0	1.8	12.1	6.4	0.5	1.6	0.7
	*CV*	0.7	0.4	0.8	0.1	0.3	0.5	0.4	0.7	0.3	0.8	0.3
Black-water	Annonaceae	84.4	72.6	2.0	52.3	12.9	0.7	4.1	6.2	0.6	2.7	1.5
	Apocynaceae	69.6	132.5	2.7	57.0	20.1	0.9	5.1	4.0	0.7	4.5	2.6
	Chrysobalanaceae	31.7	95.7	6.1	48.9	12.7	0.5	3.4	1.2	0.8	3.6	1.8
	Euphorbiaceae	7.4	78.8	1.5	53.0	10.6	0.4	1.2	2.2	0.7	4.0	1.4
	Fabaceae	36.9	105.5	9.5	58.7	20.1	0.8	3.2	2.5	0.8	4.4	2.4
	Lauraceae	38.3	72.5	1.8	51.9	14.1	0.6	3.9	1.1	0.6	1.2	1.5
	Lecythidaceae	53.1	97.9	2.9	52.1	17.3	2.1	3.9	2.4	0.7	2.6	2.6
	Malvaceae	60.5	103.0	1.8	52.2	18.1	0.7	3.9	3.4	0.6	3.6	1.6
	Melastomataceae	55.0	75.9	1.7	60.0	17.0	0.5	3.2	1.4	1.0	2.0	1.4
	Moraceae	38.7	118.4	1.8	52.8	17.9	0.7	5.3	7.8	1.2	5.0	9.0
	Myrtaceae	35.2	95.0	1.7	54.3	12.4	0.7	2.7	1.8	0.8	4.2	2.3
	Sapotaceae	35.5	69.8	7.5	53.0	13.4	0.5	4.5	1.3	0.8	2.7	2.8
	mean-value	45.0	91.5	1.8	53.7	15.6	0.7	4.3	2.8	0.7	3.3	1.8
	*CV*	0.6	0.3	0.2	0.1	0.2	0.7	0.9	0.9	0.2	0.6	0.4

### The influence of habitat on trait divergence

All functional traits except leaf chlorophyll content and lenticel density varied significantly between forest types. The likelihood ratio test was not significant for leaf phosphorus, potassium, calcium and height:diameter, indicating that both models have the same support. This means that genus does not have a strong effect on trait values (Table 5) and that habitat has a stronger effect on trait values than genus. Leaf area, specific leaf area, leaf dry matter content, leaf nitrogen and branch wood density had higher likelihood values of models considering habitat and genus effect, which indicates that the variance was explained by both habitat and genus. The marginal and conditional R^2^ differed little, especially for leaf nutrient concentration, leaf dry matter content and height:diameter ratio, which indicates that genus is not strongly affecting plant traits. A similar pattern was observed at the family level, but most of traits were explained by both habitat and family effect. The likelihood ratio test was not significant for leaf area, leaf dry matter content, leaf nitrogen and height:diameter ratio, indicating a strong effect of habitat. Specific leaf area, leaf calcium, phosphorus, potassium and lenticel density were best explained by the model considering habitat and family. The differences between marginal and conditional R^2^ differed little too, suggesting that family, as well as genus, is not strongly influencing plant trait variation beyond habitat.

**Table 5:** Variation in 11 functional traits of white- and black-water flooded forests considering pairing at genus and family level. The columns indicate β-coefficient, p-value, marginal R squared (variance explained by the fixed effect (habitat); r^2^m), conditional R squared (variance explained by the fixed effect + random effect (habitat + genus/family); r^2^c) and likelihood ratio test (p-value indicates if models differ; ratio was calculated as model_1_ (habitat + genus/family)/model_2_ (habitat)).

Plant trait	Genus						Family					
	β-coefficient	p-value	r^2^ m	r^2^ c	likelihood		β-coefficient	p-value	r^2^ m	r^2^ c	likelihood	
					ratio	p-value					ratio	p-value
LA	22.12	0.003	0.09	0.34	5.4	0.001	20.28	0.002	0.07	0.12	0.6	0.26
SLA	32.62	<0.001	0.24	0.46	5.7	<0.001	43.22	<0.01	0.20	0.32	3.4	<0.001
LDMC	-1.15	<0.001	0.67	0.72	1.9	0.05	-1.21	<0.01	0.69	0.71	0.4	0.37
Chl	1.02	0.51	0.01	0.09	0.8	0.21	-1.44	0.24	0.01	0.13	2.3	0.03
N	4.39	0.001	0.11	0.25	1.9	0.04	4.4	<0.01	0.16	0.24	1.7	0.06
P	1.23	<0.001	0.36	0.37	0	0.85	1.11	<0.01	0.33	0.43	3.7	0.006
K	7.87	<0.001	0.60	0.60	0	0.99	7.82	<0.01	0.40	0.50	4.1	0.004
Ca	2.97	<0.001	0.20	0.27	0.7	0.22	3.55	<0.01	0.20	0.32	3.6	0.007
BWD	-0.15	<0.001	0.28	0.81	33	<0.001	-0.16	<0.01	0.23	0.52	16.1	<0.001
LD	-0.73	0.1	0.00	0.15	1.4	0.09	-1.67	<0.01	0.17	0.33	5.4	0.001
HDR	-0.78	<0.001	0.62	0.62	0	0.99	-1.14	<0.01	0.48	0.49	1.7	0.06

## Discussion

We asked whether functional traits of tree species diverge between habitats differing in soil nutrient availability, and if habitat conditions are driving trait differentiation despite differences amongst genera in their evolutionary history. We found that white-water species have traits related to resource acquisition and fast-growth, while black-water species have traits related to resource conservation and persistence. Habitat effect was important to cause traits variation, even for less plastic traits.

### Traits divergence between habitat types

Plant species show different strategies to deal with rich and poor soils [48,49]. The trade-off in tissue investment that we observed in our study reflect different strategies to deal with the differences in soil nutrient availability between the two habitats. We hypothesized that congeneric species from white-water would have acquisitive trait values associated with fast-growth in a resource-rich environment whereas congeneric species from black-water would have more conservative trait values associated with persistence in a resource-poor environment. Congeneric species from white-water had indeed higher leaf area, specific leaf area and leaf nutrient concentrations which are related to high light capture, photosynthetic rates and carbon gain [19,20,38,39,50]. These characteristics result in productive plants with a short-life cycle [19,39]. White-water species had a lower height:diameter ratio. A low HDR increases plant stability against water and wind [43] and may allow white-water species to resist to disturbance from the higher water movement that is typical for white-water forests [30,51].

Congeneric species from black-water had, in contrast, higher leaf dry matter content and branch wood density. When resources are limiting, as in black-water forest, species tend to invest in strong tissues and plant longevity [36,38,41]. A small leaf area and high leaf dry matter content allow black-water species to adapt to low nutrient availability, by increasing longevity and reducing growth rate [49]. These traits are indirectly influenced by flooding and more related to plant age [52].

We also hypothesized that trait variation within forests would differ, and that in white-water forests it would be higher than in black-water forests because black-water forest is a resource-limited habitat, which imposes a strong selection filter. Trait variation in white-water forest was indeed higher than black-water forest for most traits (Table 3 and Fig 1). This result can be related to the stronger habitat filtering in black-water forest that leads to similar traits and strategies [1], or because it has a lower number of species than white-water forest, resulting in a lower trait variation. The larger variation in leaf nutrient concentrations in black-water forests may be related to stronger niche differentiation for nutrients and, hence, a larger variation in nutrient strategies in this nutrient-poor environment [53].

### The influence of habitat on trait divergence

We hypothesized that trait divergence between white- and black-water forest would be related to abiotic conditions (habitat types), indicating habitat specialization by white- water and by black-water species. Habitat type is indeed important for the divergence of labile traits between the two forests types, such as leaf dry matter content, leaf nutrient concentration and height:diameter ratio, leading to species adaptation for different habitats.

Some traits are phylogenetically conserved, but still show to some extent phenotypic plasticity. Wood density, for instance, is considered to be more conserved [54–56], but can vary in relation to environmental conditions, as flooding and soil nutrients [57–59]. Specific leaf area is very plastic in response to light availability [60], but varies very little in response to water availability [61]. In our study, wood density and specific leaf area were both affected by habitat (i.e., forest type) and by phylogeny (i.e., genus). These traits values diverged between forest types, but maintained a similar pattern within the same taxa, especially for branch wood density. For example, the genus *Mouriri* was the genus with denser wood in white-water forests (0.71 g.cm^-3^) and also the genus with denser wood in black-water forests (0.93 g.cm^-3^). When you compare the genus *Mouriri* between forest types black-water species have denser wood, but when you compare genera within habitats *Mouriri* was the genus with denser wood in both habitats (Fig 1). This result suggest that differences between habitat leads to trait variation among forests, but there is also a variation within forest type caused by differences amongst co-occurring genera.

Other traits are more labile and can respond faster to selective pressures adapting to environmental conditions, such as leaf nitrogen and leaf dry matter content [62,63]. Leaf nutrient concentration, leaf dry matter content and height:diameter ratio are related to plant growth, resource acquisition and competition [20,42], and were mainly related to habitat type (Table 5). Variation in these traits was not caused by genus identity. The convergence of these traits values within habitat, independent of genus, suggests that there is an environmental selection of these traits, and can be an ecological adaptation in response to the differences in soil conditions between black- and white-water floodplain forests.

The divergence of traits between habitats in related species can be an indication of habitat specialization to soil properties [4,12,64]. The increasing evidence of the importance of local abiotic conditions and habitat diversity for traits and species variation in the Amazonian forests [6,8], suggests that habitat specialization is an important process driving plant communities, and that trait lability probably contributes to this specialization, because it improves species adaptation to the environment [61]. Even for less labile traits, the habitat soil conditions seem to be influencing trait differentiation. The trait divergence between the congeners in theses floodplain forests seems to be, therefore, a result of habitat specialization, contributing to floristic differentiation between these areas and, hence, increasing overall species diversity.

## Conclusions

Functional traits of floodplain congeneric tree species diverge between forest types with different soil resource availability, resulting in a trade-off in tissue investment in resource acquisition and productive tissues in white-water species versus resource conservation and persistent tissues in black-water species. Both phenotypic plasticity and lability of traits are leading to habitat specialization and differentiation of floodplain tree species of white-water and black-water forests, thus contributing to a high diversity of plant species in floodplain forests.

## Supporting information

S1 Table Species of white-water and black-water forest belonging to seven genus and to twelve families. The columns indicate the phylogenetic level (genus and family) and the species belonging to each genus/ family in white-water and black-water forests.
(XLSX)

S2 Table Eleven functional trait measured of 5 individuals in seven genera at white-water and black-water forests. The columns indicate forest type, family, genera, species and functional trait values: leaf area (cm^2^), specific leaf area (cm^2^.g^-1^), leaf dry matter content (g.g^-1^), chlorophyll content (SPAD units), leaf nitrogen (g.kg^-1^), leaf phosphorus (g.kg^-1^), leaf potassium (g.kg^-1^), leaf calcium (g.kg^-1^), branch wood density (g.cm^-3^), lenticel density (count in 10 cm^-2^), and the Height:diameter ratio (m.cm^-1^).
(XLSX)

S3 Table Eleven functional trait measured in 5 individuals of twelve families at white-water and black-water forests. The columns indicate forest type, family, genera, species and functional trait values: leaf area (cm^2^), specific leaf area (cm^2^.g^-1^), leaf dry matter content (g.g^-1^), chlorophyll content (SPAD units), leaf nitrogen (g.kg^-1^), leaf phosphorus (g.kg^-1^), leaf potassium (g.kg^-1^), leaf calcium (g.kg^-1^), branch wood density (g.cm^-3^), lenticel density (count in 10 cm^-2^), and the Height:diameter ratio (m.cm^-1^).
(XLSX)
